# Interplay of the Genetic Variants and Allele Specific Methylation in the Context of a Single Human Genome Study

**DOI:** 10.3390/ijms26199641

**Published:** 2025-10-02

**Authors:** Maria D. Voronina, Olga V. Zayakina, Kseniia A. Deinichenko, Olga Sergeevna Shingalieva, Olga Y. Tsimmer, Darya A. Tarasova, Pavel Alekseevich Grebnev, Ekaterina A. Snigir, Sergey I. Mitrofanov, Vladimir S. Yudin, Anton A. Keskinov, Sergey M. Yudin, Dmitry V. Svetlichnyy, Veronika I. Skvortsova

**Affiliations:** 1 Federal State Budgetary Institution «Centre for Strategic Planning and Management of Biomedical Health Risks» of the Federal Medical and Biological Agency (Centre for Strategic Planning of the Federal Medical and Biological Agency), Pogodinskaya Street, 10, bld. 1, 119121 Moscow, Russia; 2 The Federal Medical Biological Agency (FMBA of Russia), Volokolamskoye Shosse, bld. 30, 123182 Moscow, Russia

**Keywords:** allele-specific methylation, cis-regulation, epigenetics

## Abstract

The methylation of CpG sites with 5mC mark is a dynamic epigenetic modification. However, the relationship between the methylation and the surrounding genomic sequence context remains poorly explored. Investigation of the allele methylation provides an opportunity to decipher the interplay between differences in the primary DNA sequence and epigenetic variation. Here, we performed high-coverage long-read whole-genome direct DNA sequencing of one individual using Oxford Nanopore technology. We also used Illumina whole-genome sequencing of the parental genomes in order to identify allele-specific methylation sites with a trio-binning approach. We have compared the results of the haplotype-specific methylation detection and revealed that trio binning outperformed other approaches that do not take into account parental information. Also, we analysed the cis-regulatory effects of the genomic variations for influence on CpG methylation. To this end, we have used available Deep Learning models trained on the primary DNA sequence to score the cis-regulatory potential of the genomic loci. We evaluated the functional role of the allele-specific epigenetic changes with respect to gene expression using long-read Nanopore RNA sequencing. Our analysis revealed that the frequency of SNVs near allele-specific methylation positions is approximately four times higher compared to the biallelic methylation positions. In addition, we identified that allele-specific methylation sites are more conserved and enriched at the chromatin states corresponding to bivalent promoters and enhancers. Together, these findings suggest that significant impact on methylation can be encoded in the DNA sequence context. In order to elucidate the effect of the SNVs around sites of allele-specific methylation, we applied the Deep Learning model for detection of the cis-regulatory modules and estimated the impact that a genomic variant brings with respect to changes to the regulatory activity of a DNA loci. We revealed higher cis-regulatory impact variants near differentially methylated sites that we further coupled with transcriptomic long-read sequencing results. Our investigation also highlights technical aspects of allele methylation analysis and the impact of sequencing coverage on the accuracy of genomic phasing. In particular, increasing coverage above 30X does not lead to a significant improvement in allele-specific methylation discovery, and only the addition of trio binning information significantly improves phasing. We investigated genomic variation in a single human individual and coupled computational discovery of cis-regulatory modules with allele-specific methylation (ASM) profiling. In this proof-of-concept analysis, we observed that SNPs located near methylated CpG sites on the same haplotype were enriched for sequence features suggestive of high-impact regulatory potential. This finding—derived from one deeply sequenced genome—illustrates how phased genetic and epigenetic data analyses can jointly put forward a hypotheses about the involvement of regulatory protein machinery in shaping allele-specific epigenetic states. Our investigation provides a methodological framework and candidate loci for future studies of genomic imprinting and cis-mediated epigenetic regulation in humans.

## 1. Introduction

Variations in non-coding regions of the genome have emerged as significant contributors to disease susceptibility, playing a crucial role in gene regulation and expression [1]. Unlike coding variants that typically influence disease by altering protein sequences and functions, non-coding variants are often located within enhancer regions and can affect disease risk through regulation of genes and non-coding RNA [2]. These non-coding variants can influence the binding of transcription factors, change the epigenetic profile of the DNA, and ultimately impact gene expression both locally and at a distance [3].

The advent of direct long-read DNA sequencing has opened up new avenues for investigating genetic and epigenetic variations simultaneously. By sequencing large genomic fragments, researchers can accurately discriminate between alleles and identify haplotype-specific variants within the diploid human genome. Technologies like Oxford Nanopore sequencing further facilitate the detection of epigenetic modifications, allowing for an in-depth understanding of interindividual variations in DNA methylation. These variations can be associated with disease susceptibility, and they underscore the potential for using methylation profiles as biomarkers for risk assessment, diagnosis, and prognostic predictions. A precise characterization of epigenetic differences among alleles is vital for unraveling the intricate cis-regulatory mechanisms that govern gene regulation in response to environmental factors. Allele-specific methylation (ASM) represents a key aspect of this complex regulatory landscape. ASM can occur in both imprinted loci and non-imprinted contexts, with the latter demonstrating heritability and dynamism influenced by various environmental conditions. Interestingly, while sequence-dependent ASM often arises in regions exhibiting identical primary DNA sequences, it can still have a profound effect on neighboring regulatory elements and gene expression [4].

Understanding the interplay between genetic variations, particularly single-nucleotide polymorphisms (SNPs) and allele-specific methylation is crucial for shedding light on the molecular mechanisms underpinning disease susceptibility. Recent large-scale studies have indicated that SNPs may influence the methylation levels of 34% to 45% of CpG sites, thereby affecting the functional landscape of the genome [5,6]. Moreover, mediation analyses have demonstrated that methylation can serve as a critical mechanism through which genetic risk manifests in diseases such as rheumatoid arthritis and inflammatory bowel disease [7,8].

This study aims to investigate the multifaceted relationships between genetic variants and epigenetic modifications. By unraveling these complex interactions, our research seeks not only to enhance the understanding of genetic predisposition to complex diseases but also to pave the way for novel therapeutic strategies and personalized medicine approaches. The integration of genetic and epigenetic datasets holds the potential to revolutionize our understanding of disease mechanisms and inform future research.

## 2. Results

### 2.1. Assessment of the Coverage Impact on Methylation Site Detection and Phasing Quality

In order to investigate allele-specific methylation, we have applied whole-genome sequencing with Oxford Nanopore Technology (ONT). The technology yields long and ultra long reads that allow phasing and computational estimation of the methylation status for each allele in a diploid organism [9]. Here, whole-genome ONT sequencing (WGS) was performed on PBMC obtained from the one healthy individual. The total coverage estimated for the GRCh38 human reference genome is 112X. Moreover, we also performed short-read whole-genome sequencing of the individual’s parents with the Illumina approach (paired-end reads of 150 bp), achieving 54 coverage of the paternal and maternal genomes. Thus, the ONT reads can be phased with and without parental information. One of the major questions underrepresented in the scientific literature deals with estimation of the coverage impact on the quality of methylation phasing. To address this question, we assessed the influence of sequencing depth on allele-specific detection of epigenetic modifications using subsampling (coverages of 12, 26, 79, and 112).

We analyzed epigenetic modification sites separately for each allele of the individual and compared two computational approaches for detecting allele-specific methylation signals. The first method utilized information obtained from the results of parental whole-genome sequencing, allowing allele-specific methylation assessment with trio binning. The second approach relies on the computational tools to perform phasing purely from the long-read ONT sequencing followed by quantitative detection of methylation levels for each allele.

**Figure 1:**
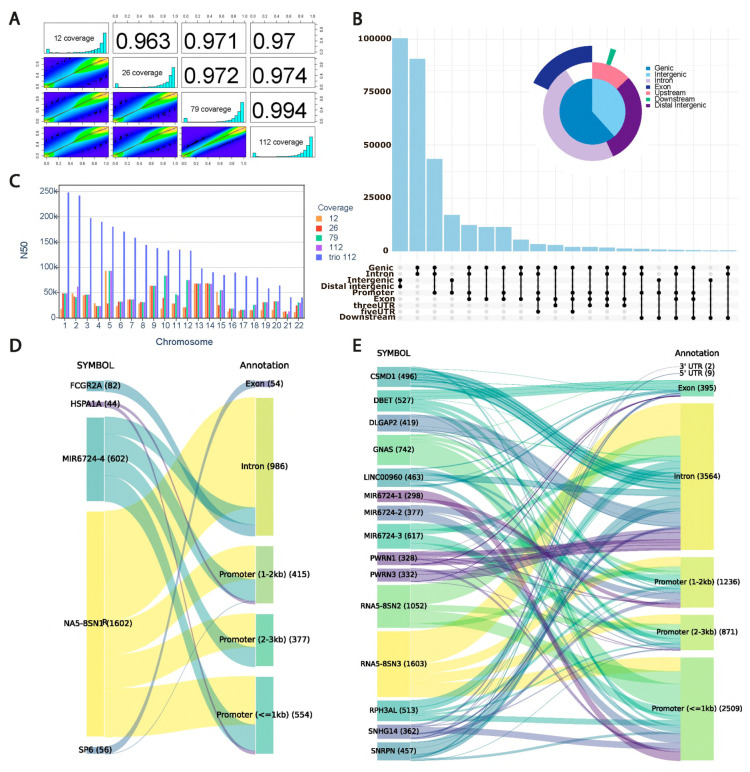
Assessment of coverage impact on methylation site detection and phasing quality. (**A**) Pearson correlation between per-site methylation frequencies detected at different coverages. (**B**) UpSet plot with distribution of the differentially methylated CpGs across genomic regions. (**C**) Comparison of the N50 phasing quality metric for haplotypes at different coverage levels for standard phasing and phasing on the parent haplotypes (trio). (**D**) Transcribed regions phasing errors annotation by genomic segments. The number of sites in each category is given in brackets. (**E**) Transcribed regions misassignment error annotation by genomic segments. The number of sites in each category is given in brackets.

Next, we estimated the impact of ONT sequencing coverage on methylation (5mC), calling for the alleles using state-of-the-art approaches [10,11,12]. Our results indicate that without trio binning, the increase of the ONT sequencing depth demonstrates high quantitative agreement of 5mC signals (Figure 1A).

Our further analysis focused on the detection of significant ASM regions. Using trio binning and 112X ONT coverage, we detected 58,470,893 methylation sites in total. However, only 308,673 (0.5%) were determined to be ASM sites. We have analyzed the distribution of ASM regions in the genome and identified that the largest fraction of the detected sites with significant methylation changes belong to the genic positions (Figure 1B). Nearly 31% belong to the promoters or gene introns. Both introns and promoters are genomic regions with high impact on gene expression regulation [13]. Considering that sites are differentially methylated, this may have consequences for the gene expression differences between alleles.

In order to estimate the impact of the ONT sequencing coverage on phasing and further quality of allele-specific methylation calling, we have estimated the N50 metric with and without trio binning. From our results indicate that a sample with coverage of 112X phased with parental information significantly outperforms the approach without trio binning (Figure 1C, Figures S1 and S2). Moreover, without parental short-reads WGS, increase of the ONT sequencing coverage above 25X does not improve N50 metric.

Identification of the correct differentially methylated sites across alleles is extremely important for biological interpretation of the epigenetic changes. To this end, we looked at the positions that are not consistently identified as differentially methylated with and without trio-binning approach due to phasing issues. Here, we consider differentially methylated CpGs identified only without trio binning as phasing errors with complete or partial allele misassignment. In the first case all reads from a genomic region are incorrectly assigned to the opposite parental haplotype, whereas for the second one only a fraction of the reads belong to the incorrectly determined haplotype. Our analysis identified that misassignment errors in transcribed regions accounted for 1.54%, while phasing errors were 0.02% of the total number of positions with observed, but not necessarily significant, differences between alleles. The majority (n = 1603) of the allele misassignments belong to the *RNAS-8SN3* gene, with prevalence of the intronic location, and 41% of all phasing errors belong to the intronic or promoter regions (Figure 1D,E). The highest number of phasing errors was observed in non-coding regions, *GNAS*, nuclear ribonucleoprotein polypeptides, *DLGAP2*, and *CSMD1* (Figure 1D,E).

Our investigation provides a high-resolution assessment of phasing-related artifacts in allele-specific methylation calling; the frequency and genomic distribution of such errors may vary across individuals due to differences in haplotype structure, genetic diversity, and local sequence complexity. Therefore, these findings should be considered as illustrative of potential technical confounders rather than as population-level estimates.

### 2.2. Genetic Variants near Allele-Specific Methylation Positions

**Figure 2:**
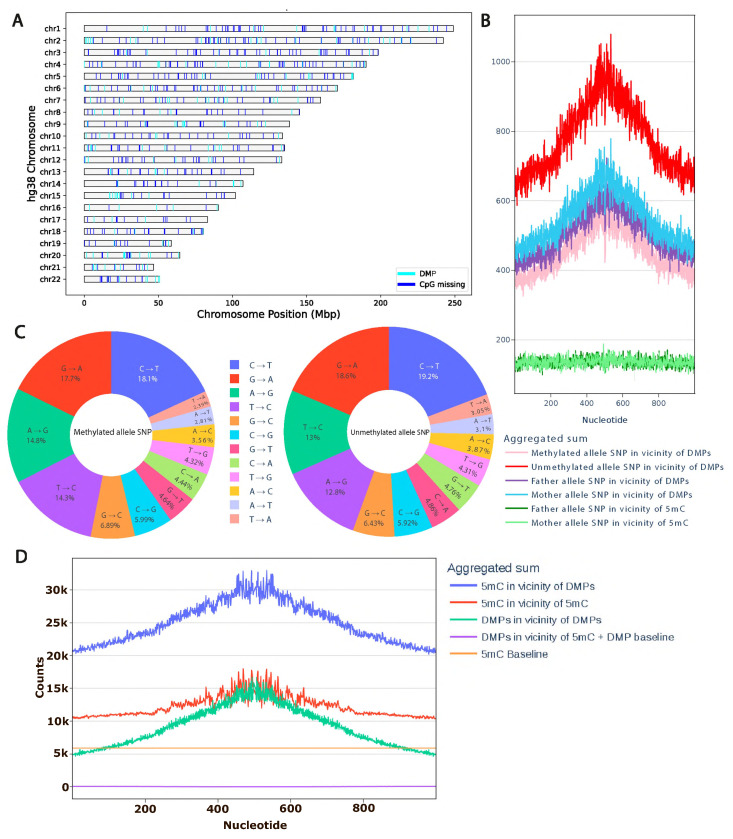
Genetic variants near allele-specific methylation positions. (**A**) Representation of differential allele-specific methylation positions caused by substitution in the CpG site among all differential allelic methylation positions in the genome. (**B**) Spatial colocalization aggregation plot of single-nucleotide genetic variants with regular and differential methylation positions centered on differential allelic methylation positions. (**C**) Comparison of single-nucleotide genetic variants near differential methylation positions on methylated and unmethylated alleles. (**D**) Spatial colocalization aggregation plot of differential methylation sites with regular methylation positions. Aggregation plots were calculated for an equal number of ASM sites and sites of normal methylation. The baseline was calculated assuming random distribution for methylation positions (orange, 5904) and differential allelic methylation (purple, 33).

We examined the genomic distribution of ASM regions and CpG sites absent in the individual relative to the hg38 reference genome. Our analysis reveals that both ASM-associated regions and missing CpG sites are attributable to individual-specific genomic variants distributed across all chromosomes, with no significant enrichment or depletion on any particular chromosome or locus (Figure 2A). Furthermore, we did not observe a systematic shift in the distance distribution between ASM sites and lost CpGs, indicating that CpG loss does not occur preferentially in the immediate vicinity of ASM signals (Figure S3).

To further investigate the interplay between genetic variation and epigenetic states, we examined the relationship between ASM and local mutation patterns flanking 5-methylcytosine (5mC) sites. Non-CpG genetic variants can modulate the activity of cis-regulatory elements by altering transcription factor binding, thereby reshaping local gene regulatory landscapes and influencing the epigenetic state of genomic loci [14]. We hypothesized that germline genetic variants in cis to CpG sites may contribute to allele-specific methylation patterns. To test this, we called high-confidence, allele-resolved germline variants from Oxford Nanopore Technologies (ONT) whole-genome sequencing data using Clair3, and quantified their density in genomic windows surrounding ASM sites. Strikingly, we observed a pronounced enrichment of single-nucleotide variants (SNVs) specifically on the hypomethylated allele—a pattern consistent across both maternal and paternal haplotypes. ASM regions exhibited a 4- to 10-fold increase in local SNV density. Critically, the majority of these variants resided on the unmethylated allele, suggesting a potential mechanistic link between local genetic variation and epigenetic asymmetry.

This finding supports a model in which cis-acting genetic variants contribute to—or are selected in association with—allele-specific epigenetic states, highlighting a non-random co-localization of genetic and epigenetic divergence in the human genome.

Importantly, trio-based phasing combined with deep sequencing coverage achieved a genome-wide phasing accuracy exceeding 99% (Figure S1). This error rate is comparable to the inherent limitations of the GRCh38 reference assembly—used here for variant calling—which contains unresolved gaps in telomeric and centromeric regions. Consequently, any residual phasing error introduced by our approach is negligible relative to the substantially larger differences in single-nucleotide variant (SNV) density observed between hypomethylated and hypermethylated allele-specific methylation (ASM) sites, as well as between ASM and bulk 5mC contexts.

We also investigated SNV signatures around ASM regions on both alleles from the angle of methylation status. In the vicinity (±500 base pairs) of these sites, C->T transitions are the most common and account for 18–19% of cases (Figure 2C). However, single-nucleotide polymorphisms directly at CpG sites account for 14% of all detected ASM. Overall, we did not identify a significant difference in substitution types between methylated and unmethylated alleles.

This observation could be explained by the clustering of ASM sites. Upon testing this hypothesis, it was found that the sites of differential methylation spatially colocalize primarily with the positions of regular methylation. This means that they predominantly occur in regions rich in methylation and do not exhibit increased clustering compared to positions of regular methylation. This suggests that the observed increase in the number of heterozygous variants of single nucleotides near the site is a significant property of the differential methylation positions themselves, rather than an artifact of their location (Figure 2D).

### 2.3. Assessment of Differences in Regulatory Activity

**Figure 3:**
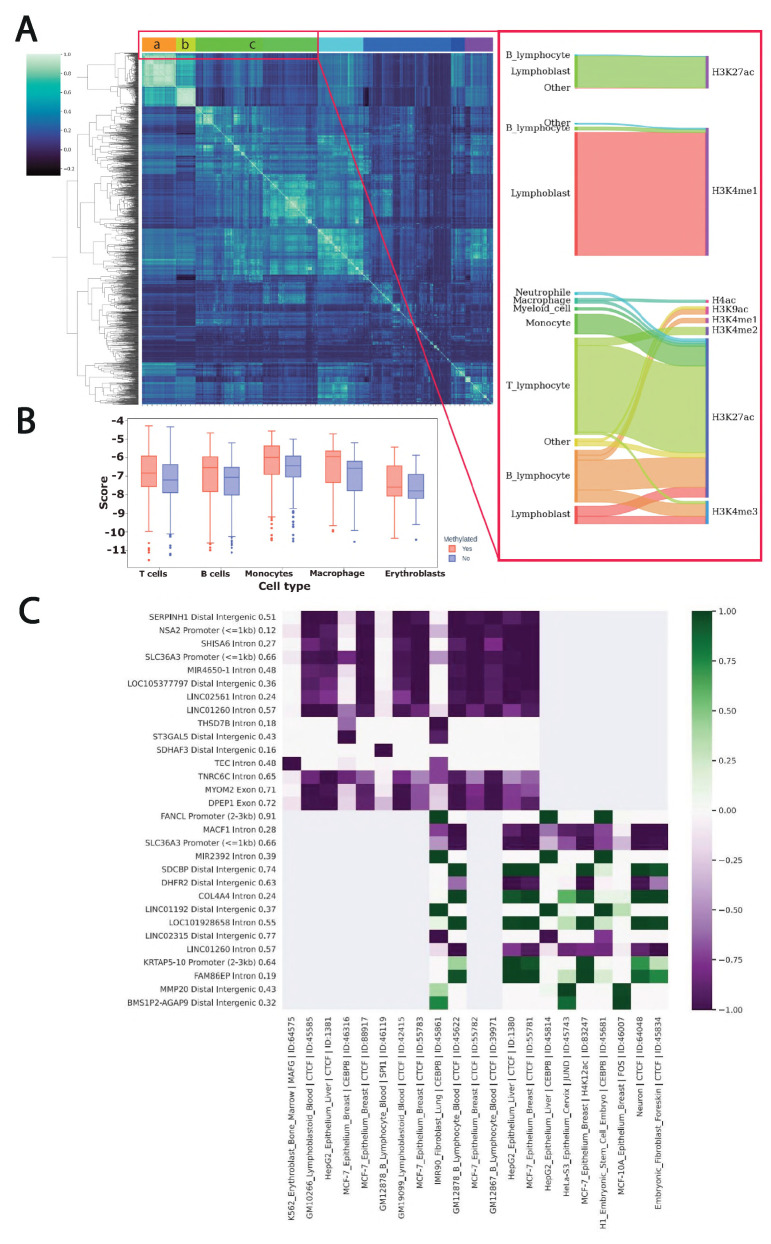
Assessment of regulatory activity at differential allele-specific methylation positions. (**A**) Pearson correlation of the 2041 most significantly differentiating patterns of regulatory activity of differentially methylated alleles. The cellular composition and epigenetic characteristics of the most homogeneous clusters are highlighted in red. (**B**) Cell type-specific comparison of Sei predictions based on methylated and unmethylated allele sequences. (**C**) Sei predictions for single-nucleotide heterozygous variants (within 500 bp from ASM) that most significantly affect regulatory functions on methylated (top 15 ASM regions) and unmethylated (bottom 15 ASM regions) alleles. The column caption is the description of the SNP position: the nearest gene, the genomic region, and the methylation delta of the nearest differential methylation site. The row caption is the Sei model name.

In the study we investigated the 5mC landscape in the blood mononuclear cells. Differences in the variant frequency around unmethylated ASM regions (Figure 2B) raises questions about the influence of the variants on the methylation of the adjacent CpG sites. Changes in the methylation occur due to the involvement of the methylase/demethylase enzymes, and attraction via protein–protein interactions can be affected by perturbation of cis-regulatory function with genomic variants. In order to assess the cis-regulatory impact of SNVs, we scored each variant with the Sei [15] model to identify the cell type/tissue where the sequence variant perturbs epigenomic features. We scored the DNA sequences of alleles centered at the ASM using Sei 1000bp. Next, we correlated scores across predictions for each of the 21,907 classes representing cell type and corresponding chromatin feature (transcription factor binding, H3K27Ac, etc.). Our analysis revealed a large cluster of the correlated outputs across all models (Figure 3A). The cluster represents models for myeloid and lymphoid cell types. We further investigated the types of chromatin modifications and identified that regulatory active histone marks (H3K27Ac, H3K4Me1, H3K4Me3) are the most perturbed features. We suggest that the predicted effect brought by the allele-specific SNVs with respect to the major histone marks of active cis-regulatory regions reflects a high impact on DNA loci function and presence of the epigenetic changes. We further investigated how the predicted regulatory impact of variants differs depending on the methylated/unmethylated status of the adjacent CpGs. To this end, we integrated scores coming from multiple models for the major blood cell populations in order to obtain a general overview and discover cell type-specific trends. The comparison revealed that variants on the methylated allele have significantly (p.adj < 0.05) higher perturbation impact (Figure 3B).

We assume that DNA variations in the vicinity of the methylated site contribute to epigenetic modification of the adjacent CpG sites. We investigated the general regulatory trend that a variant yields. To this end, we selected SNVs near the ASM on the methylated and unmethylated allele. Our analysis of the Sei predictions demonstrated the clear separation of the variants with corresponding gain of the regulatory signal for methylated alleles and loss for the unmethylated (Figure 3C). Thus, the finding additionally confirmed the high and orthogonal regulatory impact that a variant brings with respect to gene regulation. Moreover, our results indicate a strong interplay between variant-based perturbation of the regulatory function and epigenetic modifications.

### 2.4. Conservation and Distribution of the ASM Across chromHMM States and Genomic Segments

The genomic context of a methylation site critically influences the functional consequences of epigenetic variation. In this single-genome analysis, we identified a total of 308,673 differentially methylated positions exhibiting allele-specific methylation (ASM). To assess the novelty of these ASM sites, we compared them to ASM catalogs from ten previously characterized human genomes (HG002, HG005, NA19240, HG02080, HG03492, HG03098, HG01243, HG02723, HG02055, HG00733, and HG01109). Strikingly, 7.5% of our ASM sites were not observed in any of these reference individuals, suggesting the presence of individual-specific epigenetic configurations. Notably, given that our analysis is based on a single deeply sequenced genome, these findings represent proof-of-concept observations; the prevalence and conservation of such private ASM sites across the human population remain to be determined.

**Figure 4:**
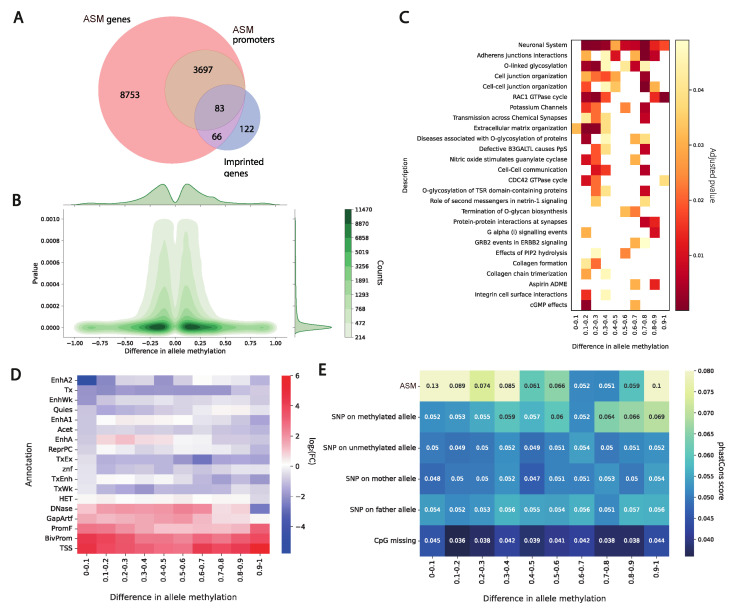
(**A**) Overlap between genes associated with ASM sites in this study and known imprinted genes from the Geneimprint database. ASM-associated genes were defined as those containing ASM sites either within the gene body or exclusively in the promoter region. (**B**) Distribution of methylation asymmetry across ASM sites. *P*-values indicate statistical significance of allelic methylation imbalance at each site. (**C**) Enrichment of ASM-associated genes in Reactome biological pathways (FDR < 0.05). (**D**) Genomic Association Tester (GAT) heatmap showing log_2_-fold enrichment of ASM sites across genomic annotations (e.g., promoters, enhancers, and repeats). Rows represent ASM site categories; columns represent genomic features. (**E**) Evolutionary conservation (phastCons score) of ASM sites compared to flanking heterozygous SNVs. Conservation is stratified by methylation state (methylated vs. unmethylated allele) and CpG-disrupting variants.

We further investigated ASM for association with biological functions and processes. First, we identified that hypo/hypermethylated sites are symmetrically distributed between alleles. From our analysis it follows that methylation differences are indeed almost equally presented in maternal and paternal chromosomes (Figure 4B). Next, we binned deltas and performed GO enrichment analysis. Our results indicate that a major trend of the associated processes and categories belongs to the functioning of the nervous system and organization of synapses (Figure 4C). Our experimental investigation has been performed on blood cells where the neural effects can barely be observed. Moreover, the individual and parents we sequenced are healthy. We can assume that the identified differences in the allele methylation are not related to pathological conditions and represent normal variation in the population.

We further evaluated ASM distribution across chromHMM [16] states of the human genome. Overall, ASM regions are enriched in the promoters, bivalent promoters, and TSS chromatin states, and the enriched states are common for all deltas of the methylation signal between alleles (Figure 4D).

We further examined the evolutionary conservation of allele-specific methylation (ASM) sites in relation to the magnitude of methylation asymmetry. Our analysis revealed a significant enrichment of single-nucleotide variants (SNVs) on the unmethylated allele (Figure 2B). Importantly, this assessment incorporated not only ASM sites themselves but also flanking heterozygous SNVs phased to each parental haplotype.

Consistent with prior work showing that ASM sites are under stronger evolutionary constraint than adjacent heterozygous variants—suggesting functional relevance and selective pressure [17]—we found that ASM sites exhibit significantly higher sequence conservation compared to surrounding SNVs (Figure 4E). This effect was markedly stronger for the methylated allele, implying that the maintenance of methylation at these loci may be subject to purifying selection.

Conversely, CpG sites lost in this individual due to genetic variants (e.g., C>T or G>A substitutions disrupting the CpG dinucleotide) showed the lowest conservation scores among all categories analyzed, supporting the interpretation that such sites are under minimal functional constraint.

### 2.5. Relationship Between Allele-Specific Expression and Promoter Methylation

In order to elucidate the relationship between allele-specific expression and allele-specific promoter methylation, we performed direct RNA-seq sequencing with ONT in three replicates (Section 4) (Figure S3). Next, we performed an estimation of allele expression followed by an analysis of differential expression between alleles with DESeq2 [18]. In total, we identified differential allele-specific expression of 24 genes, and *IFITM3*, *RPS23*, and *S100A10* also exhibited differentially methylated promoters between two alleles.

**Figure 5:**
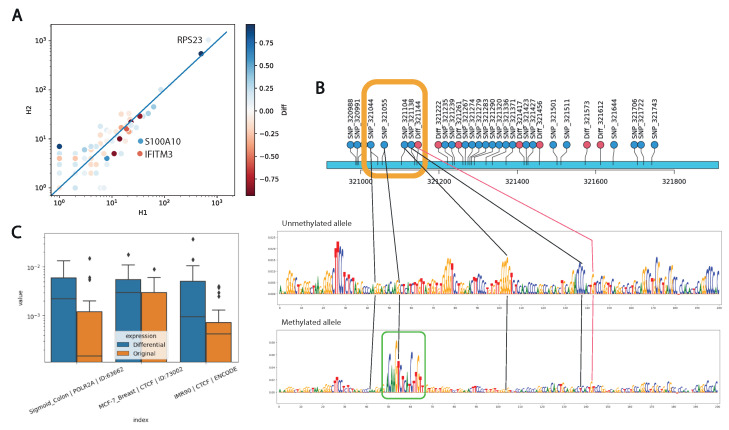
Relationship between allele-specific expression and promoter methylation. (**A**) Comparison of allelic gene expression by haplotypes (H1 and H2) and promoter methylation levels (Diff). Differentially expressed genes are labeled. (**B**) The influence of single-nucleotide genetic variants in the IFITM3 promoter region on the cis-regulatory activity calculated by the DeepLIFT method on Sei predictions for the K562_Erythroblast_Bone_Marrow | RFX1 | ID:64988 model. P53 binding sites are highlighted in green. (**C**) Sei models showing significant differences in predictions for the regulatory function of promoter regions of 24 differentially and commonly expressed genes.

We expected to see a negative correlation of allele expression with the increase in the methylation signal on an allele. In general, we have not observed a significant negative correlation for expression and epigenetic changes (Figure 5A). Furthermore, of the three differentially expressed genes, only IFITM3 represents an example of increased expression with decreased methylation of the corresponding allele. In contrast, the *S100A10* gene demonstrates increased expression with increased methylation of the promoter allele, while the *RPS23* gene demonstrates decreased expression levels with a very small difference in methylation levels.

We used the Sei neural network to analyze the individual sequences of each allele. Of all Sei models, we selected the blood model K562_Erythroblast_Bone_Marrow | RFX1 | ID: 64988 that showed the highest regulatory difference (delta = 0.111) between reference and alternative allele sequences. The impact of single-nucleotide substitutions that distinguish maternal and paternal alleles on regulatory activity is illustrated in (Figure 5B), where a significant regulatory motif TP63 is observed on the methylated allele.

To elucidate the general regulatory function of promoter regions of differentially expressed genes compared to those expressed equally on both alleles, we scored the primary DNA sequence of each allele with the Sei neural network. As a result, three models of chromatin profile were identified, showing significant differences in promoters of differentially expressed genes compared to the control group, two of which were characterized by the activity of the CTCF transcription factor (Figure 5C).

## 3. Materials and Methods

### 3.1. DNA Extraction

Genomic DNA (gDNA) from blood samples was extracted using a QIAamp DNA Mini Kit (Qiagen, Hilden, Germany) in accordance with the manufacturer’s protocol. The yield and purity of the isolated gDNA were manually determined using Quantus fluorometer (Promega, Madison, MI, USA) and NanoDrop 8000 (Thermo Fisher Scientific, Waltham, MA, USA), respectively. Only gDNA samples with absorbance ratios A260/280 of 1.7–1.9 and A230/260 of 1.8–2.2, in accordance with procedure, were selected for further analysis.

### 3.2. RNA Extraction

Total RNA from peripheral blood mononuclear cells (PBMC) was extracted using an RNeasy universal mini kit (Qiagen, Germany) in accordance with the manufacturer’s protocol. The yield and purity of the isolated RNA were manually determined using a Qubit 4 fluorometer (Thermo Fisher Scientific, USA) and NanoDrop 8000 (Thermo Fisher Scientific, USA), respectively. RNA integrity number (RIN) measurements were performed using the High-Sensitivity RNA ScreenTape on Agilent 4200 TapeStation (Agilent Technologies, Inc., Santa Clara, CA, USA). Only RNA samples with a concentration of at least 50 ng/µL, RIN of at least 7 (RIN > 7), and absorbance ratios A260/280 of 1.8–2.2 were selected for further analysis.

### 3.3. UHMW DNA Extraction

Genomic DNA from tissue samples was extracted using Nanobind UHMW DNA Extraction (Circulomics, Baltimore, MD, USA) in accordance with the manufacturer’s protocol (EXT-BLU-001). Input amount was 1.5 mL of whole blood per reaction. The presence of the isolated gDNA was manually confirmed using Quantus fluorometer (Promega, USA).

### 3.4. Nanopore Ultra-Long DNA Sequencing

For Nanopore Ultra-Long DNA sequencing, all DNA from the UHMW DNA Extraction step was used to prepare a library with the Ultra-Long DNA Sequencing Kit (SQK-ULK001) according to the manufacturer’s protocol (Version: ULK_9124_v110_revB_24Mar2021). Libraries were sequenced on a PromethION P48 (Oxford Nanopore Technologies, UK) using FLO-PRO002 flowcell.

### 3.5. Nanopore RNA Sequencing

For Nanopore sequencing, 1000 ng of total RNA was taken for each sample. Direct RNA Sequencing (SQK-RNA002) (Oxford Nanopore Technologies, Oxford, UK) was used to prepare libraries. Sample preparation was carried out according to the manufacturer’s protocol (Genomic DNA by Ligation, version DRS 9080 v1 revS_14Aug2019). Libraries were sequenced on a GridION (Oxford Nanopore Technologies, Oxford, UK); loading concentration per cell was 50 pM.

### 3.6. Illumina DNA Sequencing

A total of 150–500 ng of gDNA was used to prepare NGS (Next-Generation Sequencing) libraries. Libraries were prepared using an Illumina DNA Prep kit (Illumina, Inc., San Diego, CA, USA) according to the manufacturer’s recommendations using the Tecan Freedom EVO robotic station (Tecan, Männedorf, Switzerland). gDNA concentrations in library samples were measured using the Infinite F Nano Plus reader (Tecan, Switzerland). The size of the resulting libraries was determined with an Agilent D1000 reagent kit using the Agilent 4200 TapeStation (Agilent Technologies, Inc., Santa Clara, CA, USA). Pooling was performed automatically using a Tecan Freedom EVO robotic station (Tecan, Switzerland). Each of the 24 samples of the library pool was diluted to the final gDNA concentration of 1.5 nM prior to sequencing. Pool quality control was performed with an Agilent High Sensitivity D1000 Screen Tape reagent kit using the Agilent 4200 TapeStation (Agilent Technologies, Inc., Santa Clara, CA, USA). WGS was performed on an Illumina NovaSeq 6000 (Illumina, Inc., USA) with an S4 reagent kit (Illumina, Inc., USA) upon 300 cycles with 2 × 150 bp paired-end reads with at least 30× average depth of coverage.

### 3.7. Illumina DNA Processing

Illumina short reads were aligned to the reference using Illumina DRAGEN Bio-IT Platform v07.021.510.3.5.7, rev. 1.0. The GRCh38.d1.vd1 sequence was used as a human reference genome. The performance of the pipeline was validated with the set of high-confidence variant calls HG001, provided by the Genome In A Bottle (GIAB) consortium (v.3.3.2). The pipeline precision was 99.88%, the sensitivity was 99.78%, and, as a result, the F-score was 99.83%. The quality of generated BAM files was checked with DRAGEN, FastQC v0.11.9 [19], samtools v1.13 [20], and mosdepth v0.3.1 [21]. Minimal mean sequencing coverage for samples that passed for further analysis was 30×, Q30>85. Small genetic variants (SNP, indels up to 50 b.p.) were identified with Illumina Strelka2 v2.9.10 software with default settings [22]. Variants with the "PASS" filter were selected for further analysis.

### 3.8. Nanopore DNA Processing, Variant Calling, and Haplotype-Resolved Methylation Detection

The obtained sequencing results, FAST5 files, were processed using the Megalodon (version 2.3.4) tool (Megalodon 2.3.3 Documentation, 2023) using the default parameters according to the manual with GRCh38 as the reference genome. The model configuration file of Megalodon, res_dna_r941_prom_modbases_5mC_CpG_v001.cfg was used to detect 5mC in CpG contexts.

Single-nucleotide variants (SNVs) and small insertions/deletions were called from aligned Oxford Nanopore Technologies (ONT) long reads using Clair3, with the platform-specific model set to --platform=ont. Resulting variant calls were filtered to retain only high-confidence sites annotated with the “PASS” flag in the VCF FILTER field.

To evaluate phasing accuracy, we applied PhaseME [23] to the phased SNV set. Haplotype phasing of heterozygous variants was subsequently performed using WhatsHap (phase mode), leveraging aligned long reads (BAM) with the --ignore-read-groups parameter to ensure uniform treatment of sequencing batches.

Raw methylation calls (per-read CpG states) were processed using the methyl_call_processor module of NanoMethPhase [24]. The output was coordinate-sorted and indexed using tabix for efficient genomic querying. Finally, haplotype-specific methylomes were constructed using the phase module of NanoMethPhase, which integrates (i) phased variant calls (VCF), (ii) aligned long reads (BAM), and (iii) indexed methylation calls, all assigned to the GRCh38 reference genome. This integration enables allele-resolved quantification of CpG methylation states across phased haplotypes.

Genomic regions, SNPs, and ASM regions were annotated by intersecting the gene architecture using the R package ChIPseeker [25]. We used Reactome pathway analysis to analyze the functional enrichment of genes showing an abundance of differentially methylated positions in their promoters. Regions of ASM were tested for enrichment at gene regulatory regions using the Genomic Association Test (GAT) tool [26]. Conservation analysis of positions was performed with Phastcons (100 vertebrates) metrics from phastCons100way.UCSC.hg38 R package [27]. Chromatin-state segmentation was performed with ChromHMM v1.22 using the 15-state model. Binarized input files were generated following standard preprocessing steps.

### 3.9. Regulatory Activity Predictions

To identify sequence regulatory activities and putative enhancers, we employed Sei, a deep-learning-based framework that predicts a compendium of 21,907 chromatin profiles across more than 1300 cell lines and tissues. We investigated the regulatory activity of 1000 base pair sequences centers at the allele-specific methylation positions (+500 bp). The DNA sequences including all individual single-nucleotide variants of each allele have been scored on the methylated and unmethylated alleles. Additionally, we analyzed the regulatory activity of individual heterozygous single-nucleotide variants within those regions. Similarly, predictions of the regulatory activity of promoter regions (1000 bp from TSS) were obtained for each allele of 24 differentially expressed genes, as well as those genes that are equally expressed on both alleles. To compare regulatory differences between differential allele-specific and regular methylation, allele pairs of 1000 base pairs centered on the methylation position and containing all SNPs were selected. The enrichment analysis was based on the comparison of alleles and was performed using HOMER (v.4.10.4) [28] with default settings.

### 3.10. Motif Regulatory Analysis

Identification of significant motifs for determining the regulatory activity of a genome region was carried out by decomposing the output prediction of a neural network by backpropagating the responses of all neuron models for each feature of the input signal using the DeepLIFT package v0.6.13.0  [29]. DeepLIFT compares the activation of each neuron with a reference and assigns a score for its individual contribution to the prediction, which identifies the signature of the particular features of the input data that affect the prediction of the Sei neural network.

### 3.11. Nanopore RNA Processing

The sequencing results obtained from long reads, in the form of FAST5 files, were processed using the Guppy Basecaller software tool (version 4.4.1) [30], which employs neural network algorithms. To convert FAST5 files to FASTQ format, standard parameters were applied with quality filtering of reads using qscorefiltering–minqscore 7. Mapping to the reference transcriptome, version 41, corresponding to the GRCh38 genome version [31] used for DNA analysis, was performed using the minimap2 program [32] with the -ax map-ont parameter. Read phasing was carried out using the Whatshap software, similar to the phasing of DNA sequencing results. Differential gene expression analysis was conducted using the standard differential expression analysis procedure for the DESeq2 v1.30.0 software package in R. v4.3.1.

## 4. Discussion

Comprehending the gene expression regulation mechanism and the role of genetic variants in DNA methylation is a complex undertaking due to the multitude of factors involved in regulatory processes. High individual variability and susceptibility to individual changes complicate the use of a standard statistical approach [33]. To circumvent the influence of diverse molecular contexts in the study of regulatory activity, we investigated the impact of heterozygous genetic variants on allelic methylation in a single individual.

Single-nucleotide variants (SNVs) and small insertions/deletions were called from aligned Oxford Nanopore Technologies (ONT) long reads using Clair3, with the platform-specific model set to --platform=ont. Resulting variant calls were filtered to retain only high-confidence sites annotated with the “PASS” flag in the VCF FILTER field. We observed an approximately four-fold enrichment in the density of single-nucleotide variants (SNVs) in the vicinity of allele-specific methylation (ASM) sites compared to non-ASM (bulk) methylation sites. This enrichment supports the notion that local genetic variation plays a substantial role in shaping the epigenetic landscape, consistent with prior evidence linking cis-acting variants to allele-specific regulatory effects [34]. Notably, SNVs associated with the methylated allele exhibited stronger signatures of regulatory potential—suggesting that genetic differences may preferentially influence the establishment or maintenance of methylation on one haplotype. Importantly, as this analysis is derived from a single deeply sequenced individual, these observations represent a proof-of-concept demonstration of genotype–epigenotype coupling at base resolution rather than population-level generalizations.

Our study also complements known ASM CpGs across studied genomes, including intensively investigated known collections (HG002, HG005, NA19240, HG02080, HG03492, HG03098, HG01243, HG02723, HG02055, HG00733, and HG01109). We suggest that large-scale population studies of ASM will uncover variability across individuals and decipher the functional role of the epigenetic changes and how DNA methylation is influenced by genomic variants. We revealed 28,859 ASM sites not discovered previously in the 11 human genomes (HG002, HG005, NA19240, HG02080, HG03492, HG03098, HG01243, HG02723, HG02055, HG00733, and HG01109). However, evolutionary conservation analysis suggests that these positions exhibit higher conservation compared to nearby single-nucleotide variants pointing to functional significance and evolutionary selection [35]. Furthermore, results of the genomic association testing revealed enrichment of the ASM regions in bivalent promoters, and other studies revealed that DNA methylation has a dual effect at enhancer regions, where it is negatively associated with transcription factor binding sites but positively associated with the active H3K27ac mark globally [36]. We also found that 45% of the detected allele-specific methylation (ASM) sites overlap (Figure S4) with CpG sites previously reported as methylation quantitative trait loci (meQTLs) in large-scale epigenomic studies [6].

The study analyzed the relationship between allele-specific expression and allele-specific promoter methylation in total blood expression and suggested a negative relationship between allele methylation levels and gene expression, but no significant correlation was established. Studying the contribution of individual nucleotides and methylation patterns to the regulation of gene expression, we suggest that the key factor is the availability of the transcription factor binding site, which is strongly influenced by the presence of a nucleotide sequence motif and its methylation status. Also, the positions of single-nucleotide substitutions near a methylated CpG site tend to be more conserved compared to those near unmethylated sites. However, in the case of IFITM3, the emergence of a TP53 binding site due to an SNP on a methylated allele, known to enhance IFITM3 expression following P53 addition in vitro, results in higher expression from the methylated allele compared to the unmethylated allele without this SNP. Therefore, the accessibility of the transcription factor plays a crucial role in gene expression regulation, regardless of the specific molecular mechanism involved. This leads to a regulatory mechanism of gene expression where the contribution of genetic differences in alleles is adaptively balanced by methylation patterns. Furthermore, from the perspective of this hypothesis, the implications of methylation loss observed during aging become clearer [37].

To gather additional insights into the allele-specific regulatory activity, we performed motif discovery to identify altered binding sites near positions of common and differential allele-specific methylation. This effort revealed no significant differences in motifs associated with regions of common methylation. However, motifs for EFL-1 factors regulating GTPase activity and ribosome binding, as well as NFKB1 and TP53 factors involved in numerous immune processes and tumorigenesis, were detected in regions of allele-specific methylation. This indicates possible involvement of the transcription factors in the cis-regulatory activity of the loci.

In addition, our results also shed light on the technical aspects of such studies. Our analysis demonstrated that increasing long-read coverage beyond 30X did not yield significant improvements in phasing quality, underscoring the importance of triophasing as the best approach currently. Moreover, we observed phasing errors in high-coverage samples that lacked triophasing, including incorrect allele assignments, and partial haplotype assignments with higher error rates in noncoding regions and several specific gene loci.

Our study has several interrelated limitations that temper the interpretation and generalizability of our findings. First, the expression—ASM correlation analysis is restricted to only 24 genes that simultaneously exhibited robust allelic expression imbalance and high-confidence phased methylation data. This small, highly selected set limits the biological scope of our conclusions, which should be regarded as exploratory and hypothesis-generating rather than representative of genome-wide regulatory principles. Moreover, in the absence of phenotypic, longitudinal, or functional validation data, we cannot assign clinical or regulatory significance to the observed epigenetic states—even for variants annotated as “benign.” Second, technical aspects of our analytical pipeline introduce potential biases. Methylation calling and regulatory impact predictions relied on deep learning models trained on heterogeneous tissue sources, which may not generalize well to novel or uncharacterized cell types. Although our use of Megalodon was appropriate for Oxford Nanopore R9.4.1 chemistry, current best practices recommend the Dorado + Remora pipeline for both R9.4.1 and R10+ chemistries to achieve higher basecalling and modification detection accuracy and ensure compatibility with evolving community standards. Third, all analyses are based on a single parent–offspring triad. Consequently, the ASM patterns we describe reflect a unique combination of genetic and epigenetic features in one individual and cannot be extrapolated to broader populations. While long-read sequencing of this genome provides unprecedented insight into haplotype-resolved epigenetic regulation, population-level patterns of allele-specific methylation—and their interplay with genetic variation—require validation in diverse, multi-ethnic cohorts with integrated genomic, epigenomic, and phenotypic data. Taken together, these biological, technical, and population-level constraints underscore the preliminary nature of our work and highlight the need for replication in larger, well-annotated cohorts using standardized, up-to-date analytical frameworks.

## Data Availability

Raw fastq files with whole-genome sequencing data cannot be shared publicly because there are restrictions on the availability of patient samples. Data collection was performed in July 2021.

## Supplementary Materials

The following supporting information can be downloaded at: https://www.mdpi.com/article/10.3390/ijms26199641/s1.

## Abbreviations

5mC	5-methylcytosine
ASM	Allele-Specific Methylation
BAM	Binary Alignment/Map
ChIP	Chromatin Immunoprecipitation
CTCF	CCCTC-Binding Factor
CpG	Cytosine-phosphate-Guanine dinucleotide
DESeq2	Differential Gene Expression Analysis based on the Negative Binomial Distribution
	(v1.30.0)
DeepLIFT	Deep Learning Important FeaTures
DNA	Deoxyribonucleic Acid
DRAGEN	Dynamic Read Analysis for GENomics
FDR	False Discovery Rate
F-score	Harmonic mean of precision and recall
FAST5	Oxford Nanopore raw signal file format
FASTQ	File format for sequences with quality scores
gDNA	Genomic DNA
GIAB	Genome In A Bottle
GO	Gene Ontology
GAT	Genomic Association Tester
H3K27Ac	Histone H3 lysine 27 acetylation
H3K4Me1	Histone H3 lysine 4 monomethylation
H3K4Me3	Histone H3 lysine 4 trimethylation
hg38	Human Genome Build 38
indel	Insertion/Deletion
NGS	Next-Generation Sequencing
N50	Contig or haplotype length metric
ONT	Oxford Nanopore Technologies
PBMC	Peripheral Blood Mononuclear Cells
Q30	Quality score threshold (error probability = 1/1000)
RIN	RNA Integrity Number
RNA	Ribonucleic Acid
Sei	Sequence-based regulatory activity inference model
SNP	Single-Nucleotide Polymorphism
SNV	Single-Nucleotide Variant
TSS	Transcription Start Site
TF	Transcription Factor
UHMW	Ultra-High Molecular Weight
VCF	Variant Call Format
WGS	Whole-Genome Sequencing
