# Supplementary material for: Interplay of the Genetic Variants and Allele Specific Methylation in the Context of a Single Human Genome Study
Source: Int J Mol Sci. 2025 Oct 2;26(19):9641. doi: 10.3390/ijms26199641 (PMC12525282; doi:10.3390/ijms26199641)
Supplement: Supplementary file 1 [file ijms-26-09641-s001.zip › ijms-3835370-supplementary.pdf]

Supplement

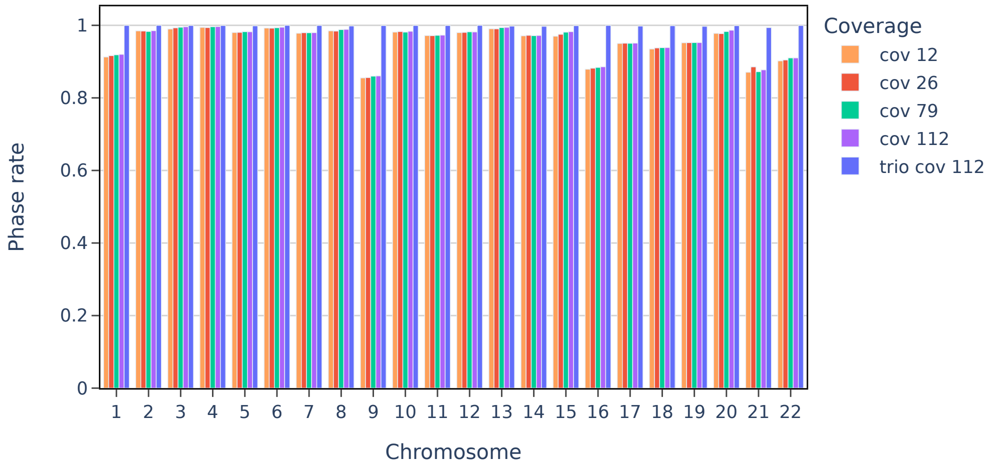

Figure S1: Comparison of the ratio of the phasing block total length to the difference in position between the last and first SNP of the chromosome for haplotypes at different coverage levels.

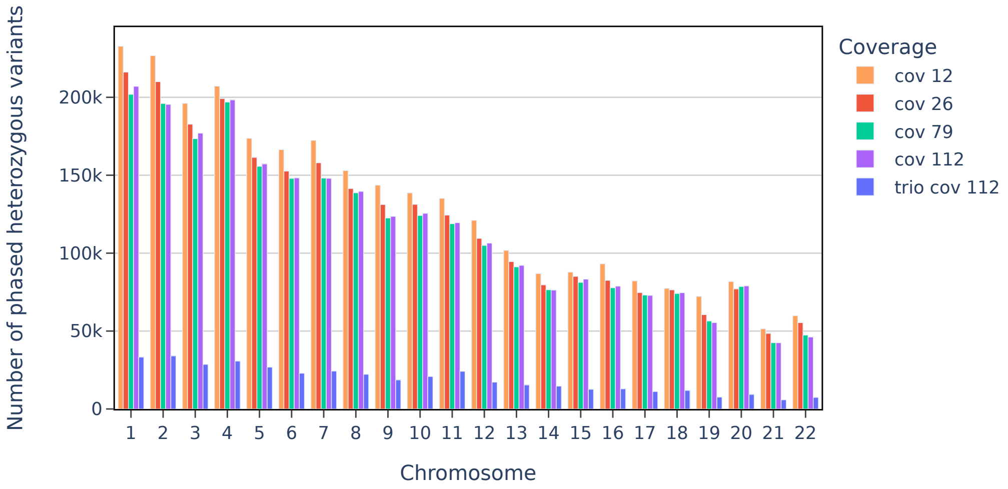

Figure S2: Comparison of the number of phased heterozygous variants for haplotypes at different coverage levels.

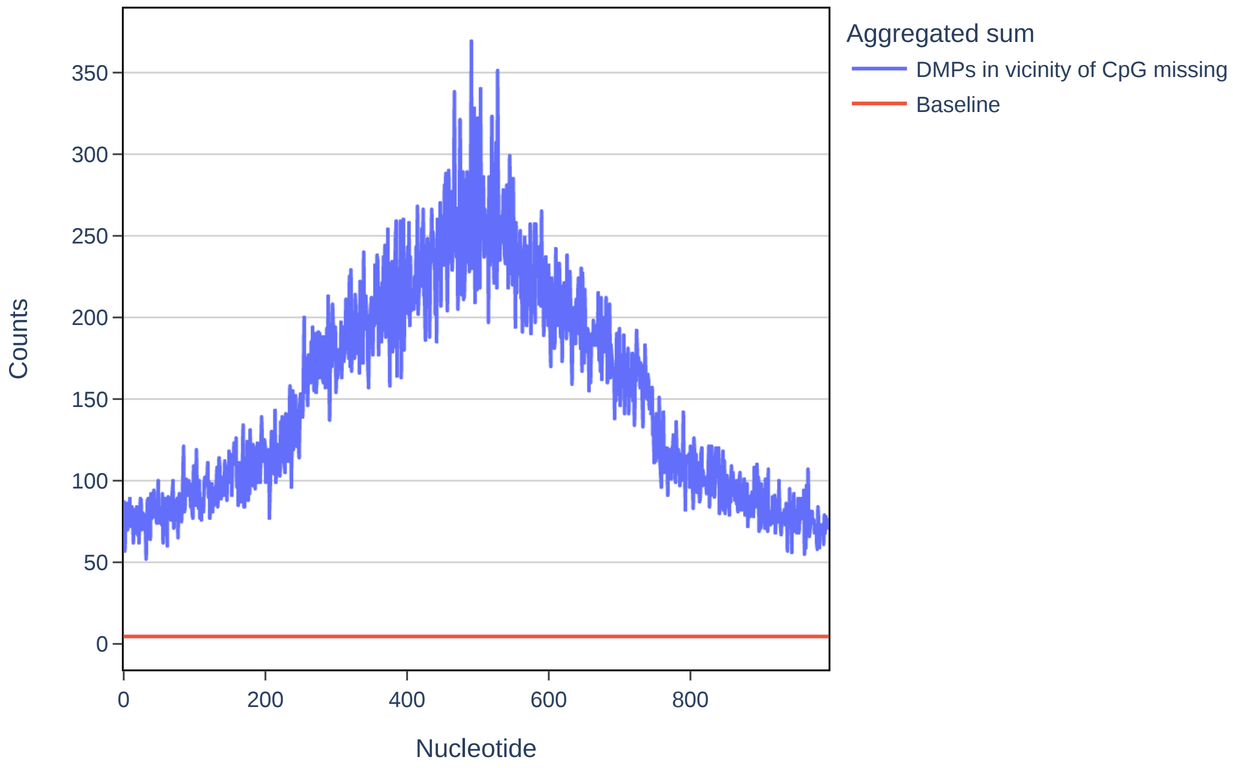

Figure S3: Spatial colocalization aggregation plot of CpG missing and differential methylation positions centered on CpG missing positions compared to random baseline.

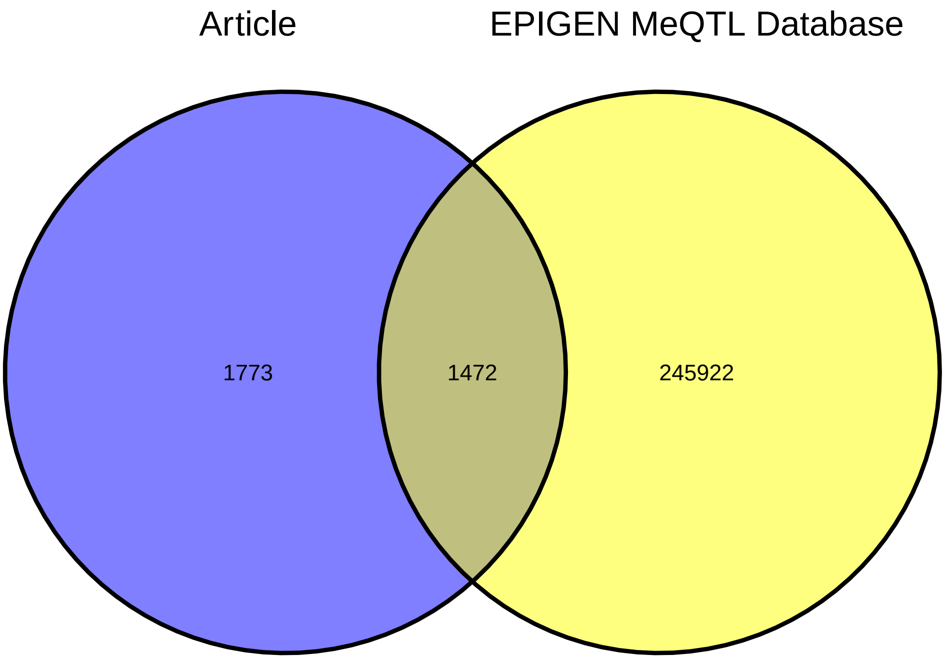

Figure S4: Overlap of the ASM sites covered by EPIC 850K arrays with EPIGEN MeQTL Database.
